# Treatment of acetabular fractures in older patients-introduction of a new implant for primary total hip arthroplasty

**DOI:** 10.1007/s00402-017-2649-3

**Published:** 2017-02-28

**Authors:** H. Resch, D. Krappinger, P. Moroder, A. Auffarth, M. Blauth, J. Becker

**Affiliations:** 1 Department of Traumatology and Sports Injuries, Paracelsus Medical University Salzburg, Muellner Hauptstr. 48, 5020 Salzburg, Austria; 2 Department of Traumatology and Sports Injuries, Medical University Innsbruck, Innsbruck, Austria

**Keywords:** Acetabular fracture, Total hip arthroplasty

## Abstract

**Background:**

Fractures of the acetabulum in younger patients are commonly treated by open reduction and internal fixation. For elderly patients, stable primary total hip arthroplasty with the advantage of immediate postoperative mobilization might be the adequate treatment. For this purpose, a sufficiently stable fixation of the acetabular component is required.

**Materials and Methods:**

Between August 2009 and 2014, 30 cases were reported in which all patients underwent total hip arthroplasty additionally to a customized implant designed as an antiprotrusion cage. Inclusion criteria were an acetabular fracture with or without a previous hemiarthroplasty, age above 65 years, and pre-injury mobility dependent on a walking frame at the most. The median age was 79.9 years (65–92), and of 30 fractures, 25 were primary acetabular fractures (83%), four periprosthetic acetabular fractures (14%), and one non-union after a failed ORIF (3%).

**Results:**

The average time from injury to surgery was 9.4 days (3–23) and 295 days for the non-union case. Mean time of surgery was 154.4 min (range 100 to 303). In 21 cases (70%), mobilization with full weight bearing was possible within the first 10 days. Six patients died before the follow-up examination 3 and 6 months after surgery, while 24 patients underwent radiologic examination showing consolidated fractures in bi-plane radiographs. In 9 patients, additional CT scan was performed which confirmed the radiographical results. 13 had regained their pre-injury level of mobility including the non-union case. Only one patient did not regain independent mobility. Four complications were recognized with necessary surgical revision (one prosthetic head dislocation, one pelvic cement leakage, one femoral shaft fracture, and one infected hematoma).

**Conclusion:**

The presented cage provides the possibility of early mobilization with full weight bearing which represents a valuable addition to the treatment spectrum in this challenging patient group.

## Introduction

The current treatment standard for displaced acetabular fractures is open reduction and internal fixation (ORIF). ORIF treatment aims to achieve anatomical reduction and stable fixation in order to allow early patient mobilization in form of touchdown or partial weight bearing [1, 2]. As the number of patients with osteoporotic bone condition is increasing [3], the typical injury scenario for acetabular fractures has shifted from younger patients involved in high-velocity accidents toward elderly patients with low-impact trauma [3–5]. While the typical fracture pattern in younger patients usually involves the posterior column and posterior wall, the typical fracture pattern observed in the older cohort is a fracture of the anterior column involving the quadrilateral plate with concomitant central dislocation of the femoral head [4]. This change has brought up new challenges to the ORIF treatment approach. In some cases, it can be difficult to achieve anatomic reduction and stable fixation especially when treating patients with osteoporotic bone quality [2, 6], which is often associated with a higher degree of comminution, impaction and cartilage damage [2, 6–8]. As elderly patients often suffer from several co-morbidities and limited physiological tolerance, a surgical intervention of long duration and subsequent limited mobility due to restricted weight bearing represents a considerable health risk.

**Fig. 1:**
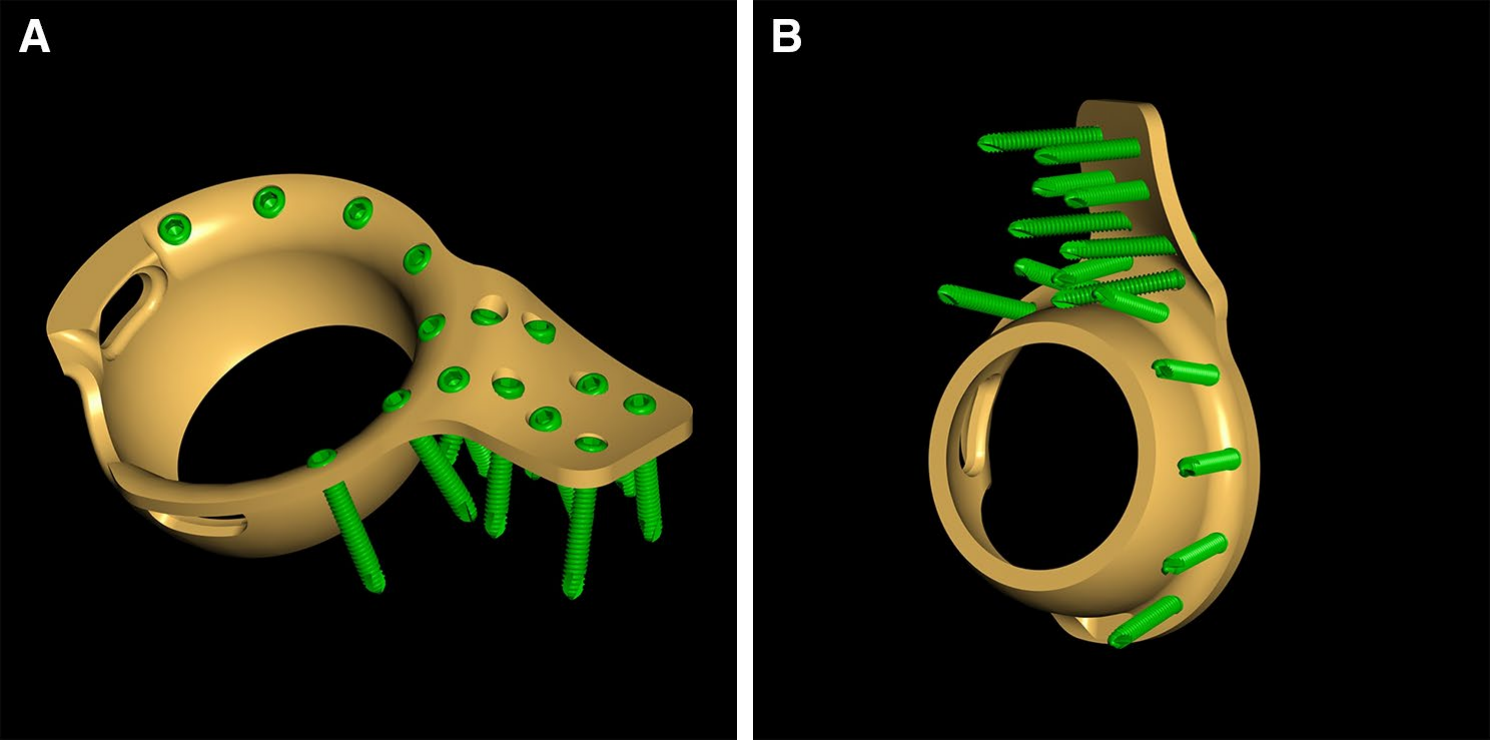
Customized acetabulum Roof-Reinforcement Plate showing the outer (**a**) and inner (**b**) surface with srews

These considerations favor the use of primary total hip arthroplasty (THA) with the potential advantage of a reduced surgery time, limited blood loss, stable fixation, and the possibility of immediate postoperative mobilization with full weight bearing. In the literature, several attempts with THA treatment in acute fracture cases were reported [9–13]. A major problem for most of these techniques was to establish stable conditions for the acetabular component [14]. Some of them used a cabling reinforcement technique [9], some others an antiprotrusion cage [10], and others plating systems in combination with THA [11, 12]. The custom-built roof-reinforcement plate was designed in an attempt to provide a solid basis for the fixation of the acetabular component by fixation of a fin of the cage at the intact iliac bone (Fig. 1a, b). The goal of this study is to describe the novel-customized implant and technique of fixation and report on first clinical and radiological results.

## Materials and methods

In this retrospective case series, we identified 30 cases of patients who were treated with the custom made roof-reinforcement plate and hip arthroplasty using the below-described technique at 3 level-one and 1 level-two trauma center between October 2009 and March 2014. Inclusion criteria were an acetabular fracture with or without a previous hemiarthroplasty, age above 65 years, osteoporotic fracture as identified by a low-energy trauma (e.g., fall from a standing height), significant marginal impaction and pretraumatic mobility on a walking frame at the most. All patients sustained a fall from a simple height except for four. Three felt from a higher level and one patient suffered from a motor vehicle accident. Of 30 patients, only six patients were below the age of 70 years, and the indications for THA were central femoral head dislocation without fracture of neither the anterior nor the posterior column in one case, a true two-column fracture with marginal impaction in another case, and a non-union after a failed ORIF treatment. Of the three remaining patients, one patient sustained an anterior column fracture with associated femoral neck fracture and two other patients suffered from a T-type fracture with concomitant multiple impactions. Acetabular fractures were classified according to Letournel [15]. Perioperatively, the days until definitive treatment, the duration of the procedure in minutes, and postoperative mobilization were recorded. In addition to the routine check-ups, bi-plane radiographs of the pelvis were taken at 3 and 6 months follow-up with accompanying CT scan if required.

### Blood transfusion management

According to our individual hospital transfusion regime for all patients, hemoglobin levels were measured pre- and postoperatively. Beyond that, intraoperative hemoglobin levels were recorded by blood gas analysis providing continuous measuring. All patients with hemoglobin level less than 10 g/dl and a central venous oxygen saturation (ScvO2) below 80% received intraoperative blood transfusion (350 ml each). Patients with hemoglobin levels less than 8 g/dl obtained blood transfusion as well. Furthermore, if symptoms of anemia occurred postoperatively, blood transfusion was administered [16].

### The implant

The custom-built Roof-Reinforcement Plate 3.5 (DePuy, Synthes, Bettlach, Switzerland) has an outer diameter of 50 mm and an inner diameter of 48 mm, which is designed for cemented cups with an outer diameter of 46 or 48 mm. On the topside, the ring of the implant is extended by a fin, which holds 8 angle stable 3.5 mm screws aiming in divergent directions in order to optimize primary stability. The implant comes in one size with different versions of the fin for the right and left hip. The fin is anatomically shaped to fit the acetabular roof and anterior or middle part of the iliac bone. The ring itself can hold seven 3.5 mm angle stable screws for fixation to the anterior column, posterior column, and the acetabular roof (Fig. 1a, b).

### Surgical technique

**Fig. 2:**
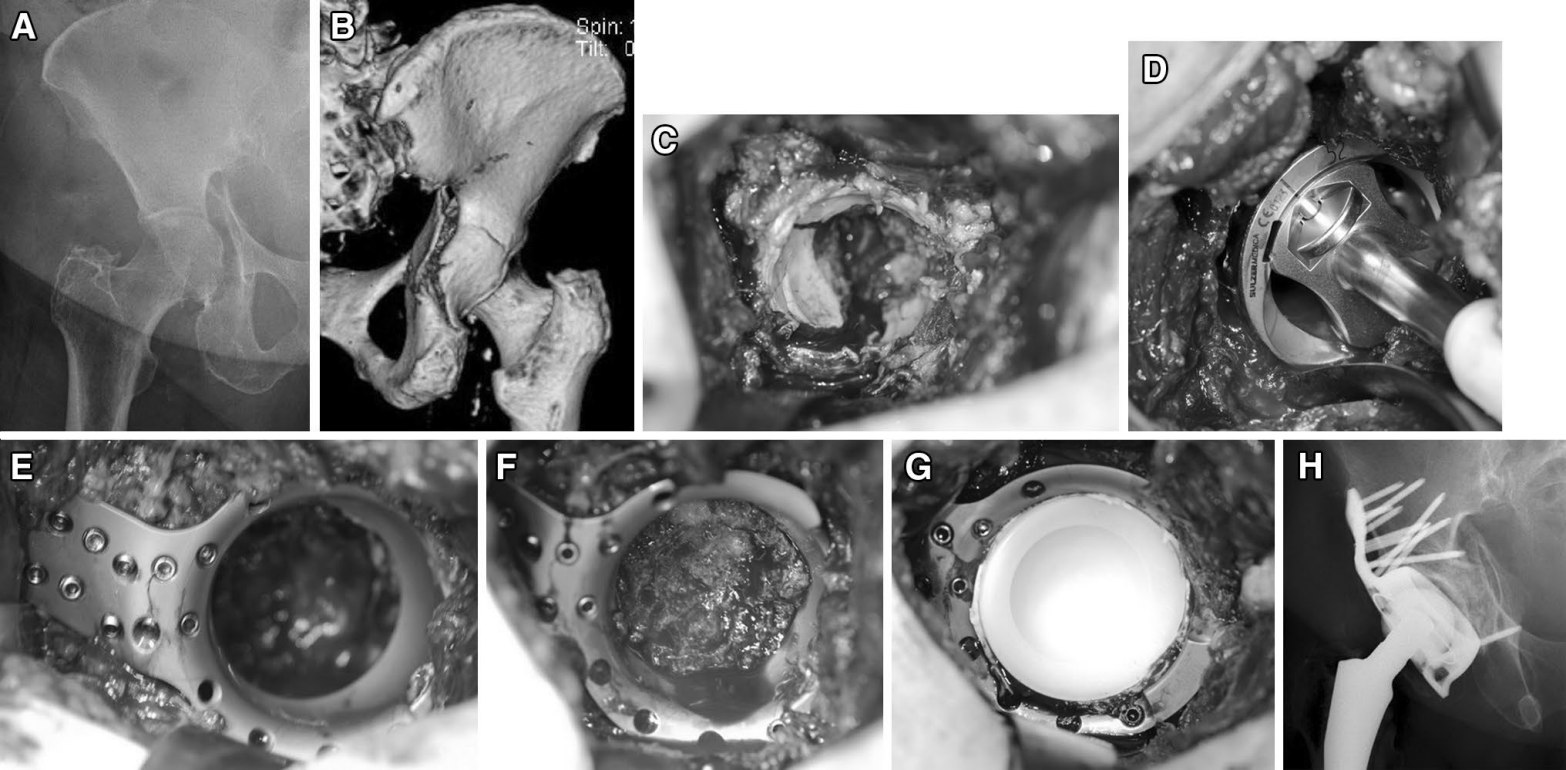
**a** Preoperative a.p. X-ray of a transverse acetabular fracture on the right side in a 79-year-old female. **b** Posterior view of a 3D-CT scan. **c** Intraoperative appearance of fracture. **d** Reaming of the fractured acetabulum up to 52 mm in diameter. **e** Custom-built Roof-Reinforcement Plate fixed with angle stable 3.5 mm screws. **f** Bone graft taken from the femoral head padding the cavity of the acetabulum. **g** Cemented 48 mm polyethylen inlay. **h** Postoperative a.p. X-ray

Under general anesthesia, the patient is placed in supine position. In contrast to the direct lateral transgluteal (Hardinge) approach, which is the standard approach for total hip replacement at our department, the anterolateral (Watson-Jones) approach is preferred for the implantation of the customized implant. This approach provides better access to the anterior and middle aspect of the iliac bone. The incision is started 2.5 cm posteriorly and distally to the anterior superior iliac spine. It is then curved distally and posteriorly to the greater trochanter and extended 5 cm distally along the shaft of the femur. The interval between the tensor fascia latae, the gluteus medius muscle, and the vastus lateralis is then identified and opened. The capsule is exposed and opened by a T-shaped incision. A femoral neck osteotomy is performed using an oscillating saw. The following surgical steps are described on the basis of a clinical case of a 79-year-old female with acetabular fracture on the right side (Fig. 2a, b). Retractors are placed anteriorly, posteriorly, and inferiorly to optimize visualization of the acetabulum. The entire capsule is removed in order to provide good exposure of the acetabulum (Fig. 2c). Next, the cartilage is removed with a sharp spoon. Regardless of the fracture type, the socket is reamed starting with a 44 mm reamer and increasing the diameter up to 52 mm (Fig. 2d). 4–5 cm of the anterosuperior aspect of the acetabular roof is exposed for positioning of the fin. The custom-built acetabulum roof-reinforcement plate is introduced without prior reduction of the fracture. The fin is fixed to the iliac bone using angle stable screws (Fig. 2e). Depending on the type of fracture and fracture level, appropriate screws around the ring are inserted. In case of an anterior column fracture, the fin is placed more posteriorly to improve the screws’ grip in cortical bone. The femoral head is used to harvest bone chips, which are placed at the bottom of the acetabulum to improve bony healing and prevent cement entrance into the pelvis (Fig. 2f). In the case of a periprosthetic fracture where no femoral head is available, a Prolene^®^ mesh-graft (Ethicon, Johnson & Johnson Medical, Norderstedt, Germany) is fixed with a number of sutures to cover the ring´s inner aperture in order to avoid cement leakage into the pelvis. A polyethylene cup of 46 or 48 mm diameter is cemented into the metal cage (Fig. 2g). Subsequently, the femoral component is implanted in a typical manner. Figure 2h shows the postoperative appearance on an a.p. X-ray view.

Postoperatively, early mobilization with full weight bearing is started within the first days after the procedure with the aid of a walking frame. In patients with a true two-column fracture where only some of the screws can be placed in stable iliac bone, partial weight bearing is recommended at least initially.

### Ethical Committee

The study was submitted and approved by the local ethical committee. The Committee has not raised concerns regarding the use of the described custom build reinforcement plate.

### Statistical analysis

This study represents a descriptive analysis of our selected patient cohort. Data are presented as mean and percentage.

## Results

Of the 30 patients included in the study, 15 were women. The median age was 79.9 years (65–92). All patients had tripped and fallen while walking on even ground except for three patients with a level fall and another involved in a motor vehicle accident (MVA). We identified 25 primary acetabular fractures (83%), four periprosthetic acetabular fractures (14%), and one non-union after a failed ORIF (3%) (Table 1). For the primary cases and the periprosthetic fractures, the average time from injury to surgery was 9.4 days (3–23). The non-union case was revised 295 days after primary ORIF treatment. Mean time of surgery was 154.4 min ranging from 100 to 303 min. The average preoperative hemoglobin level was 11.4 g/dl (9.4–14.8) and decreased by a mean of 2.0 g/dl (+0.8 to −4.8) to the average postoperative level of 9.4 g/dl (7.4–12.0). 20 patients (66.6%) required an average of 1.2 units of blood transfusions (350 ml per unit) intraoperatively and 18 patients (60.0%) needed a blood transfusion of 2–6 units postoperatively (Table 2).

**Table 1:** 30 patients with fracture of the acetabulum

Patient	Age	Sex	Trauma	Type	Classification
1. BJ	70	M	Fall	Primary	T-Type
2. BA	65	F	Level fall	Primary	Central disloc^a^
3. GF	80	F	Fall	Primary	T-type
4. KS	69	F	MVA	Primary	Both column
5. RK	69	F	Fall	Primary	T-type
6. ZM	87	F	Fall	Periprosthetic	Transverse
7. WM	90	M	Fall	Primary	Ant. column
8. SM	87	F	Fall	Primary	T-type
9. TM	88	F	Fall	Periprosthetic	Both column
10. FA	88	M	Fall	Primary	Ant. column
11. ME	84	M	Level fall	Primary	Ant. column + post hemitr
12. SA	68	F	Fall	Non-union	Both column
13. EJ	92	M	Fall	Periprosthetic	Central disloc^a^
14. TJ	86	M	Fall	Primary	Central disloc^a^
15. BF	88	M	Fall	Primary	Ant. column + post hemitr
16. DA	89	F	Fall	Primary	Ant. column
17. WM	80	F	Fall	Primary	Ant. column + post hemitr
18. FP	76	F	Fall	Primary	Both column
19. HE	78	F	Fall	Periprosthetic	Transverse
20. EF	83	M	Fall	Primary	Transverse
21. BW	67	M	Level fall	Primary	T-type
22. OF	82	M	Fall	Primary	Transverse
23. JF	91	M	Fall	Primary	Transverse
24. RA	65	F	Fall	Primary	Ant columnl
25. RM	88	F	Fall	Primary	Transverse
26. NS	79	M	Fall	Primary	Ant column + post hemitr
27. SW	87	M	Fall	Primary	Post wall
28. ZH	73	M	Fall	Primary	Ant coumn + post hemitr
29. NJ	73	M	Fall	Primary	Transverse
30. HA	76	F	Fall	Primary	Transverse

*MVA* Motor Vehicle Accident

^a^Central dislocation of the femoral head without fracture of neither the anterior nor the posterior column (not classifiable)

**Table 2:** ASA and Hemoglobin

Patient	ASA	Hb preop	Hb 1st postop day	Blood units intraop	Blood units postop
1	1	12.2	9.8	0	0
2	2	12.8	8.3	2	4
3	3	12.1	8.2	1	3
4	1	11.5	9.8	2	3
5	1	12.8	8.5	0	2
6	3	10.8	8.7	0	4
7	3	14.7	9.9	0	2
8	3	11.0	8.5	2	2
9	4	11.7	10.4	1	4
10	4	14.8	10.5	0	0
11	3	9.9	10.7	2	0
12	4	12.7	10.8	0	2
13	4	9.4	8.8	2	0
14	4	11.0	11.6	1	0
15	4	11.7	9.7	2	3
16	4	11.9	8.0	2	3
17	3	11.0	8.8	2	0
18	3	11.8	9.7	0	3
19	3	11.5	12.0	2	4
20	3	10.0	7.9	2	0
21	3	9.7	8.5	1	3
22	3	9.6	7.4	2	6
23	4	10.0	8.0	2	0
24	3	10.4	9.2	2	2
25	3	11.0	9.2	0	3
26	3	9.6	10.3	2	0
27	2	12.7	10.6	0	0
28	3	10.7	9.5	2	3
29	3	12.9	10.3	0	0
30	3	10.5	9.3	2	0

In 21 cases (70%), mobilization with full weight bearing was possible within the first 10 postoperative days. Four patients with both columns fractured started with partial weight bearing as far as they could, within the first postoperative week, and were further mobilized with full weight bearing beginning with the 21st day. Two patients had an additional fracture (femoral shaft fracture on same side and an additional undisplaced acetabulum fracture on the other side). In the remaining 3 patients, general health condition did not allow early mobilization.

One patient died within 24 h after the procedure. Five more patients with the age of 87, 88, 90, 91, and 92 years died within 6 months after surgery due to cardiac failure.

Of the 24 patients available for the 6 months follow-up, 13 had regained their pre-injury level of mobility, which was the ability to walk without any aid in five cases, independent mobilization with cane in 7 cases, and walking frame in one. The patient who had been treated for a non-union of a previously plated acetabular fracture was also able to walk without any aid at 6 months follow-up even though preoperatively she needed a cane. Nine patients had regained independent mobility, but required using a cane (5 patients), a walking frame (3 patients), or a crutch (1 patient) as walking aid, which preoperatively they did not. One patient did not regain independent mobility (Table 3). Radiologic follow-up with bi-plane radiographs showed that all fractures were consolidated within 3 and 6 months after the procedure. In 9 of the 24 cases, an additional CT scan was performed 6 months postoperatively and confirmed fracture consolidation and no loosening signs in all cases.

**Table 3:** Postoperative mobility

Patient	Pre-injury	3 months	6 months	FWB (days)
1	Free^a^	Free	Free	3
2	Free	Free	Free	21
3	Cane	Crutches	Cane	3
4	Free	Free	Free	21
5	Free	Crutches	Cane	3
6	WF	^†^	^†^	^†^
7	Cane	^†^	^†^	8
8	Cane	WF	WF	3
9	Cane	Cane	Cane	4
10	Cane	Cane	Cane	3
11	Free	WF	Cane	4
12	Cane	WF	Free	21
13	Cane	^†^	^†^	5
14	Cane	Cane	Cane	3
15	Cane	^†^	^†^	4
16	Free	Cane	Cane	3
17	Cane	WF	Cane	3
18	Cane	WF	WF	21
19	Cane	Cane	Cane	3
20	WF	WF	WF	8
21	Free	Free	Free	7
22	Cane	WF	WF	28 (fem sh fx)
23	WF	WC	^†^	0
24	Free	Cane	Cane	18 (add. acet Fx other side)
25	Cane	Cane	Cane	3
26	Cane	WC	WC	No
27	Free	Cane	^†^	6
28	Free	Free	Free	9
29	Free	One crutch	One crutch	28
30	Free	Cane	Cane	6

*FWB* Full Weight Bearing, *WF* Walking Frame, *WC* Wheel Chair

^†^These patient could not be examined for further data inquiry due to unexpected death

^a^Free Independent without any aid

## Discussion

This study introduces the custom-made roof-reinforcement plate as an operative treatment option for elderly patients with displaced acetabular fractures. Due to its stable construct, 70% of the patients could be fully mobilized within 10 days postoperatively. In total, 47% of all patients (*n* = 14) regained their pre-injury mobility level within the follow-up period. Since geriatric patients are rarely able to adhere partial weight-bearing, non-operative treatment should be avoided in this patient population because of the implemented immobilization resulting in decrease of bone metabolism [17] and exacerbation of possible co-morbidities. Therefore, surgery remains an adequate therapeutic option to treat older patients with acetabular fractures even though Letournel and Judet [18] and Matta [2] mentioned that age has an unfavorable effect on radiological [3, 6] and clinical outcome after conventional open surgical procedures.

For most acetabular fracture patterns, plate osteosynthesis is still seen as the gold standard. Due to longer operation time combined with several complications like hernias, thrombosis, and lesions of the femoral vessels using the ilioinguinal approach [2, 3, 19–21], minimal invasive techniques were introduced more recently [22–27]. Ruchholtz et al. presented a new two-incision minimal invasive technique (TIMI) for displaced acetabular fractures with a minimal follow-up time of 12 months with promising results [24]. However, his consecutive case series is difficult to compare with our results as only 14 of the 26 patients (53.8%) were older than 65 years. Unfortunately, no postoperative mobilization management was mentioned. Thus it is not clear when the patients were allowed for partial or full weight bearing. Another minimal invasive technique also published recently is the Pararectus approach of Keel et al. [22, 23]. Also, this procedure showed very promising results. In this series, only 48.1% of the patients were older than 60 years. The reported mean operation time is higher compared to our case series [23]. Due to different age of the patients’ series, comparison is difficult. We suggest that at least most of the patients might have been fit enough for partial weight bearing postoperatively. Co-morbidities may not have played the same role as it was in our series. In comparison to other published techniques with THA in the acute phase the cabling technique of Mears [9] and Mouhsine [28], only toe touch partial weight bearing for 6 weeks was allowed. Some component migration was noted in both series. No component migration was reported by Rickman et al. [12], although early full weight bearing was allowed to their patients. Due to additional rigid fixation, stable conditions could be achieved for the acetabular component. Compared to our technique, additional surgical time is needed for plating via the Stoppa approach including changeover time for the Kocher-Langenbeck approach. The most similar surgical technique compared to ours is the primary total hip replacement with a reinforcement ring (Burch-Schneider-Ring) and autologous bone grafting at the socket in 14 cases of displaced acetabular fractures [10]. In our series, all patients were allowed for partial weight bearing for the first 6 weeks postoperatively, and all fractures showed bony consolidation at follow-up. However, case series with a higher patient population and long-term follow-up are needed.

The typical osteoporotic acetabular fracture pattern involves the anterior column and the quadrilateral plate [1, 4] which according to the German Pelvic Multicenter study group results in particularly high rates of posttraumatic osteoarthritis [29]. Therefore, primary arthroplasty seems to be a reasonable approach for the treatment of acetabular fractures especially in elderly and old patients. The newly introduced implant is easy to insert while operating, and it is designed to provide high primary stability. Fixation of the anterior column can be performed by anterior screws but does not enhance primary stability. Due to this design, immediate postoperative full weight bearing even in advanced osteoporosis is allowed. In our experience, only one size of cage has shown to be necessary. In cases of a smaller acetabulum, no problems occurred while reaming up to 52 mm. Also, in cases of bigger diameters, bone grafting was performed, and no major problems appeared.

According to our results, all fractures showed bony consolidation around the implant even though no reduction of the fracture was performed. Fracture gaps were merely filled with bone chips harvested from the femoral head, which were then impacted through insertion of the cage. However, bone grafting was not possible in patients with previous hip replacement. Even in these cases, the fractures showed bony consolidation at follow-up. In contrast to a recent report of osteosynthesis and primary hip replacement [11], no secondary fracture dislocations or sintering of the implants have been observed in our patient population. In nine of the 24 patients, a CT scan was performed about 6 month postoperatively. In all cases, the CT scan confirmed the fracture healing around the cage as already suggested by conventional radiographs. All types of fractures commonly found in elderly patients with osteoporotic bone could be satisfactorily treated with the new implant including four patients with two-column fractures (no part of the acetabulum is attached to the axial skeleton). Due to the placement of multiple screws at various angulations through the fin, in all cases, at least some of the screws could be positioned in intact iliac bone (shown by postoperative CT scan).

In all patients, surgery could be performed within a reasonable time of about 154 min, which is shorter than reported elsewhere (Andersen 282 min, Saxer 164 min, Rickman 193 min) [11, 12, 27]. Regarding the perioperative blood management, our results did not differ from the reported rates of intraoperative and postoperative allogeneic blood transfusion of 58.3 and 48% for ORIF of acetabular fractures. The reported postoperative hemoglobin difference after total hip arthroplasty is around 3.5–4.0 g/dl [30]. The average postoperative hemoglobin difference in our case series was approximately 2 g/dl which combined with the average intraoperative transfusion of 1.2 units of blood equals approximately, 3.6 g/dl according to Pierson [31] and therefore does not differ from the results for primary total hip arthroplasty.

According to our treatment paradigm, mobilization was started immediately after the suction drains were removed. 21 patients were mobilized with full weight bearing within the first 10 days after surgery. The four patients with two-column fractures were mobilized with only partial weight bearing for the first 3 weeks for reasons mentioned earlier. In one patient, a fracture of the femoral shaft occurred during insertion of the prosthetic stem requiring cerclage fixation with delayed full weight bearing, and in another patient, an undisplaced acetabulum fracture of the other side did not allow early mobilization. In the remaining three patients, early mobilization could not be achieved due to restricted general health condition.

Whereas the patients in this series being nearly 80 years old on average, we record 6 deaths in the first 6 months after surgery due to cardiac failure. All 6 patients were at least 87 years old and three of them had 90 years or more (Table 2). Three of them were already mobilized by cane or walking frame before they died. Rickman et al. reported a mortality rate of 14%, but the average age in their series was 77 years [12].

We identified an overall complication rate of 13% (*n* = 4). In one patient, a dislocation of the prosthetic head occurred 3 months after surgery. We believe that poor positioning of the cemented cup inside the cage might have caused the dislocation. The second complication was cement leakage into the pelvis in a patient with a periprosthetic acetabular fracture. In this patient, no bone grafting was possible which normally would have sealed the pathway into the pelvis. Even though this was just a radiological finding without any clinical consequences for the patient, after this case, we started using a Prolene^®^ mesh-graft (Ethicon, Johnson&Johnson Medical, Norderstedt, Germany) in periprosthetic fracture cases where no femoral head is available for bone grafting. The third complication was a fracture of femoral shaft during insertion of the prosthetic stem. The fourth complication was a postoperative infected hematoma, which was evacuated with subsequent healing 3 weeks after primary surgery.

Overall, we observed very satisfying results, especially regarding early postoperative mobilization rate and pre- to postfracture mobility level. Rickman et al. presented a higher mobilization rate of 100% in 7 days compared to ours and described his postfracture mobility as independent, but still mostly requiring walking aids. However, in our case series out of 24 patients available for follow-up, 23 patients were independent mobile of which 9 patients required a walking aid [19]. The other 13 patients reached the same postfracture level of mobility they had before. Only one patient did not reach an independent mobility level in our patient cohort. Nevertheless, higher patient population and long-term follow-ups are needed to better compare such case series.

A limitation of this study is the relatively small number of patients combined with the loss of follow-up of six patients who died due to severe preexisting co-morbidities. Additionally, the retrospective study design per se carries the risk of missing information. A further limitation of this study is that not all patients underwent a CT scan 6 months after surgery to analyze fracture healing and detect potential loosening of the implant; however, all patients underwent at least bi-plane radiography in addition to the clinical investigation.

## Conclusion

Clinical decision-making needs to be individualized according to specific requirements (fracture pattern, co-morbidities, etc.). Based on our results, the custom-built roof-reinforcement plate designed for treatment of displaced acetabular fractures with poor bone quality represents a valuable addition to the treatment spectrum in this challenging patient group. Due to the specific design, early full weight-bearing mobilization seems to be a promising benefit of this implant and technique. However, careful patient selection, preoperative planning, and workup are required. Furthermore, long-term results should be evaluated.
